# The Wide Spectrum of Presentations of Cytomegalovirus Infection in Immunocompetent Hosts: An Exhaustive Narrative Review

**DOI:** 10.3390/pathogens13080667

**Published:** 2024-08-07

**Authors:** Ami Schattner

**Affiliations:** The Faculty of Medicine, Hebrew University Hadassah Medical School, Ein Kerem, Jerusalem 91120, Israel; amischatt@gmail.com

**Keywords:** cytomegalovirus, human infections, immunocompetent host, diagnosis

## Abstract

CMV is a ubiquitous DNA virus that establishes infection and results in 40–100% seropositivity. Viral replication occurs following an acquired primary infection (or reinfection) or by the reactivation of life-long latency. In immunocompetent patients, CMV infection is mostly asymptomatic or mild and self-limited. However, an extensive review of the literature published up to April 2024 reveals that despite immunocompetence, CMV can cause a very large variety of clinical syndromes in any part of the gastrointestinal tract (the most common pattern), the central or peripheral nervous system, and the eyes, as well as hematological, pulmonary, cardiac, and cutaneous disease. Not uncommonly, more than one system is involved, and though the disease is often self-limited, treatment with intravenous ganciclovir or oral valganciclovir may be required, and in isolated cases, fatalities may occur. Thus, a potential CMV infection should be considered in the differential of myriad syndromes in non-immunocompromised patients. Associated systemic symptoms (fever, sweats, and weight loss), lymphocytosis, and hepatitis are not uncommon and can be a useful clue. Some populations, such as critically ill patients in intensive care, pregnant women, elderly patients, and those with inflammatory bowel disease, may be more susceptible. Moreover, the potential of past, latent CMV infection (i.e., CMV seropositivity) to be associated with significant cardiovascular morbidity and all-cause mortality years later is intriguing and requires further study. All these data indicate the outstanding importance of developing a vaccine against CMV, which hopefully will become available in the foreseeable future. Meanwhile, a solid diagnosis of active CMV infection can be quickly established (or ruled out) by widely available serology tests and PCR amplification, and clinicians in all disciplines need to be more aware of the diverse guises of CMV infection and remember to consider it in any host, including an immunocompetent one.

## 1. Introduction and Background

Herpesviridae are a large family of DNA viruses containing over 130 different species, classified into three major subfamilies: alpha, beta, and gamma herpesviridae [1]. However, only nine herpersvirus types primarily infect humans. These are herpes simplex viruses 1 (HSV-1) and 2 (HSV-2) and varicella zoster virus (VZV), of alpha herpesviridae; in the beta herpesviridae subfamily, human herpes viruses 6A, 6B (HHV-6), and 7 (HHV-7), of the roseolovirus genus, and cytomegalovirus (CMV); and among gamma Herpesviridae, Epstein–Barr virus (EBV) and Kaposi’s sarcoma-associated herpesvirus (human herpesvirus-8, HHV-8). Of these nine types, five are extremely widespread, affecting many millions of people worldwide. They include HSV, VZV, EBV, and CMV.

All herpesviridae share a common, unique four-component structure, which contains a core of double-stranded DNA genome encoding 100–200 genes enclosed in a protein cage (capsid), surrounded by an amorphous protein coat (tegument), and encased in a glycoprotein-bearing lipid bilayer membrane (envelope) [1]. During infection, viral envelope glycoproteins bind to cell-membrane receptors and the virion is internalized so that viral DNA reaches the host cell nucleus. Within the infected cell’s nucleus, the transcription of lytic viral genes, genome replication, and capsid assembly occur. Importantly, after a primary lytic infection, which is either asymptomatic or associated with a self-limited illness, herpesviruses establish life-long latent infections and persist in the host cells indefinitely. These latent infections are asymptomatic but can become reactivated and cause substantial morbidity in human hosts. The interactions of CMV with the immune system and the mechanisms underlying CMV latency and reactivation are complex and remain incompletely understood. Clearly, herpesviruses must evade the immune system in the early phases of lytic infection. Two established pathways in CMV infection involve the virus-induced downregulation of the major histocompatibility complex (MHC) I and II [2,3,4] and the inhibition of the synthesis of myriad pro-inflammatory cytokines through the viral human IL-10 homolog cmvIL-10 [5,6]. T cells are especially important in controlling CMV replication but fail to completely eliminate the virus. Indeed, CMV-specific CD4+ and CD8+ T cell responses have been demonstrated in immunocompetent hosts [7,8] but do not prevent virus latency. Apparently, latency is established in myeloid progenitor cells after an initial lytic gene expression is silenced through epigenetic repression [9]. Latently, CMV-infected monocytes then disseminate the virus to many distant organs. Spontaneous reactivation can be induced after many years of latency by immunosuppression associated with disease or its treatment but also by common triggers such as inflammation or infection and by cellular aging/damage [10].

Thus, clinically overt CMV infections can present in one of three different forms of productive: a primary, acute CMV infection in a CMV-naïve individual; reinfection with another strain despite natural immunity; and, commonly, reactivation of latent CMV acquired previously, with the resolution of the primary infection but occult virus persistence in myriad reservoirs [10]. The sites of CMV latency are primarily peripheral blood monocytes and bone marrow hematopoietic progenitor cells, but the liver and spleen, lungs, and other tissues are additional identified sites of latency [10]. CMV seroprevalence in the adult population is very high worldwide, estimated at 40–100% in the general population depending on geographic region and socioeconomic factors. Higher rates are associated with increasing age, female gender, developing countries, and lower socioeconomic status [11,12]. Primary infection often occurs during childhood or adolescence, but primary infection, reinfection, and reactivation of latent infection are all substantially prevalent. For example, in a prospective cohort study of adolescent girls in the US, Finland, and Mexico who were followed for three years, baseline CMV seroprevalence was 58%. Primary infection occurred in 14.8% of seronegative girls. Among seropositive girls, 5.9% had ≥4-fold increase in anti-CMV antibodies, and 23.9% shed CMV DNA in urine [13]. In another prospective study of seropositive women followed over a mean of 35 months, as many as one-third had CMV reinfection [14]. In another study, CMV DNAemia (24% at baseline, 52% over follow-up) and viruria (39% at baseline, 83% over follow-up) were quite commonly encountered despite seropositivity [15]. However, this remarkable prevalence notwithstanding, CMV infections are mostly asymptomatic or mildly symptomatic, demonstrating a benign, self-limited course. It is mostly in immunocompromised patients (in particular, solid organ or hematopoietic cell transplant recipients, HIV-infected patients, and following cancer chemotherapy) that CMV reactivation or infection is considered in the context of serious, severe disease [16,17]. Another vulnerable population is that of pregnant women, since CMV infection can be associated with fetal transmission. Congenital CMV infection affects up to 40,000 infants per year in the US and can result in serious neurodevelopmental disabilities in offspring [18,19]. In contrast, clinicians often fail to consider CMV as the cause of a severe illness in an apparently immunocompetent, often previously healthy patient. To increase clinicians’ awareness of the large spectrum of presentations of severe CMV-associated disease not related to a recognized immunodeficiency state, and thus facilitate timely diagnosis and treatment, we have undertaken a comprehensive review of the literature.

## 2. Methods

PubMed was searched using the MeSH terms “cytomegalovirus”, “CMV”, “CMV infection” AND immunocompetent (or: nonimmunocompromised, or nonimmunosuppressed) for abstracted publications in the English language on “humans AND adults” (search terms) since the database’s inception and up to April 2024. Severe disease was defined as an illness requiring hospitalization, but cases demanding intensive ambulatory investigations were included. Immunocompetency was defined as the absence of either a primary congenital immunodeficiency or acquired immunodeficiency (such as human immunodeficiency virus [HIV] infection, solid organ or hematological transplantation, malnutrition, active cancer, autoimmune disease) or immunosuppressive treatment. Patients with inflammatory bowel disease (IBD) were not excluded unless they had received immunosuppressive medications in the 3 months preceding CMV infection. All qualifying articles were reviewed, and in addition, their reference lists were scanned to identify and include additional relevant studies.

## 3. Results

**Figure 1:**
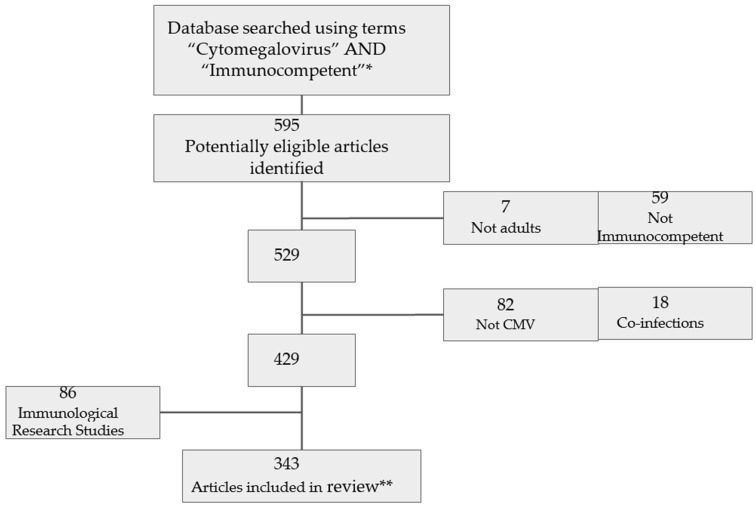
Flowchart of the studies included in this review. * Exact search terms were “cytomegalovirus”, “CMV”, “CMV infection” AND immunocompetent or: nonimmunocompromised, or nonimmunosuppressed. ** Including 9 retrospective case series of severe CMV (4–115 patients, median 16 patients) and 2 series based on a review of the literature (performed 1997 and 2008) [see text].

Overall, 595 articles were identified by our search. However, 252 were excluded (e.g., immunology-focused non-clinical research, studies on non-immunocompetent patients, such as post-transplant or HIV-infected patients, etc.), as presented in Figure 1.

The remaining 343 articles were included in this review together with additional relevant publications found in their listed references. The majority reported on single case studies, with several notable case series. Nine case series not limited to any single organ system were found (n = 4 to 115 patients, median 16 patients), all of them retrospective studies. Two additional studies reported on cases collected from the literature (n = 34 and n = 290), the last one was published in 2008 (see below). All selected articles satisfied the established criteria for the diagnosis of active CMV [20,21]. This was usually based on serologic criteria. CMV-specific IgM antibodies are sensitive markers that confirm active infection, but they cannot distinguish primary infection from reactivation. In addition, in IgM-negative cases, at least a fourfold increase in IgG titers was considered to support ongoing infection. In pregnant women, low avidity of CMV IgG (i.e., the strength of IgG binding to antigenic epitopes) was taken to indicate primary CMV infection within the preceding 3 months [22]. Since 1992 [23], diagnosis has often been established by molecular amplification methods—the detection of CMV DNA by real-time polymerase chain reaction (PCR) in the blood, urine, or other samples. Often, data from both methods (after 1992) were provided. Serological tests may be initially negative, highlighting the importance of clinical awareness [24]. In some reports, tissue was available for pathological examination and immunohistochemistry, especially when gastroscopy/colonoscopy or surgery were performed. When tissue was obtained and histopathology and immunohistochemical analysis showed CMV, this was also considered proof of the diagnosis, as was a CMV cell culture, which was not performed often [21].

Twelve comprehensive studies (not limited to a single organ system) were devoted to severe CMV in immunocompetent patients. Two of them were reviews of the literature. The first one identified only 34 cases in the world literature up to 1996 [25]. The last one was published in 2008 and identified 89 articles reporting on 290 immunocompetent adults [26]. As mentioned, the number of reports has greatly increased since (in our review of the literature, 212/343). All the other publications are case series based on retrospective chart reviews reporting on between 5 and 115 hospitalized patients (median 13–14 patients) published between 2001 and 2017 [27,28,29,30,31,32].

These studies and our review of the literature reveal that gastrointestinal involvement is the most common severe form of CMV infection in immunocompetent patients and CMV-induced CNS disorders constitute the second most frequent manifestations of severe CMV, with hematological disease coming third [27,30]. Each may take many forms and when first presenting to the general practitioner (primary care provider), may be misleading and erroneously interpreted as a nonspecific viral illness (in 38%) [28], fever of unknown origin [FUO] [22,33] or malignancy. Fortunately, the marked presenting systemic symptoms of fever + marked fatigue and lassitude + night sweats + weight loss, which are almost invariably present in different combinations, may in conjunction with several recurring laboratory findings strongly suggest CMV infection if present:

1. Lymphocytosis/atypical lymphocytes (median 54% in several case series) was noted in most of the patients’ blood counts and peripheral blood smears [32]. While sensitivity is high, it is less than 100%, and in one study, it was absent in 1:3 patients [27].
2. Liver enzyme disturbances [LEDs]—predominantly increases in hepatocellular enzymes (alanine aminotransferase [SGPT], aspartate aminotransferase [SGOT], lactate dehydrogenase [LDH])—were reported in 9:10 patients in many series [27,30]. Thus, LEDs are more common and can be found *without* lymphocytosis in severe acute CMV among immunocompetent patients [27].
3. Absence of an alternative explanation, which should always be considered.

In addition, the patients’ age was often between 20 and 59 years old in 90% of patients [28] and was a mean 42.5/median 44 years in another group [30]. However, case series often focus on one form of severe CMV (fever of unknown origin, venous thrombosis) [30,32,33] or are too small [31] to capture and reflect the spectrum of the various systems potentially affected in severe CMV infections.

This was better reported in the two reviews that highlighted the importance of central nervous system (CNS) involvement, the common occurrence of multiple organ involvement by CMV, and the generally good prognosis of these patients [25,26].

### 3.1. The Diverse Clinical Spectrum of Primary, Reactivated, or Reinfected CMV

Our own major finding in an extensive review of the literature relates to the very large spectrum of disorders in very many systems that severe CMV infection can take in immunocompetent patients, often at presentation. These are summarized in Table 1 (gastrointestinal and liver/pancreatic disease); Table 2 (neurologic and ocular disease); Table 3 (hematological involvement); Table 4 (pulmonary and cardiac involvement); Table 5 (severe systemic and mucocutaneous involvement and that of other varied types); and Table 6 (postulated long-term adverse consequences associated with past CMV infection). The latter (Table 6) are not reported manifestations of active CMV infection (Table 1, Table 2, Table 3, Table 4 and Table 5), but rather significant associations identified between past CMV infection (an utterly common event in most populations) and future health-related consequences.

The essence of each organ system’s involvement will now be separately, although briefly, discussed.

### 3.2. Severe CMV-Associated Gastroenterological, Hepatic, and Pancreatic Manifestations in Immunocompetent Hosts

#### 3.2.1. Gastroenterological

Several clear and striking findings emerge when looking at the ample literature on CMV-induced gastrointestinal disease. First, judging from the large number of case reports identified in this review, the gastrointestinal tract (GIT) is the most frequently affected organ in severe illness caused by CMV among immunologically competent patients [27]. In a large review of 290 immunocompetent patients with severe CMV infection, GIT involvement constituted the most frequent manifestation [26]. Second, from the esophagus to the rectum, any part of the GIT can be directly involved by CMV, and this can be very convincingly demonstrated in tissue biopsies [34,35]. However, CMV infection of the colon and rectum is the most common, observed in 84% of 56 patients vs. 25% experiencing the involvement of the stomach or esophagus and 23% experiencing the involvement of the ileum [36]. In this study, the sum of over a 100% indicates that combined area infection is possible and common. Colon-dominant disease (in 70% of 86 immunocompetent patients) was also found in other studies [37]. Third, unlike any other body system affected by CMV, a diagnosis of GIT CMV can be strongly supported by biopsies obtained by upper endoscopy or colonoscopy (in addition to the usual serology and PCR-based methods). Pathology reveals that a mass can be found but is relatively unusual, affecting approximately 1:10 patients. The two predominant lesions anywhere in the GIT are ulcers (mucosal ulceration) and inflammatory mucosa. In fact, ulcers were found in 82.5–92% of patients [37]. Patients’ presenting symptoms and the range of complications are directly derived from these pathologies.

Thus, in the colon-dominant group of patients, hematochezia and diarrhea are the predominant symptoms. They are generally not to be found in the upper GIT-dominant group, whose major complaints are melena/hematemesis and odynophagia as well as nausea, vomiting, and dysphagia [37]. Abdominal pain is commonly noted in both types of GIT involvement (but is especially frequent in the small-bowel-dominant group, followed by the colon-dominant group). These case series and literature reviews also reveal several unique characteristics of GIT CMV patients vs. severely ill patients with other systems’ involvement [35,36,37]. Most patients are older, with mean ages 67–70 years in one series and 73 years in another [36,37]. In fact, 67/86 patients were aged 60 years or more (78%). Accordingly, metabolic comorbidities and atherosclerotic diseases were commonly associated with CMV infection. Paradoxically perhaps, their presentation was often severe, so that 1:5 patients required admission to an intensive care unit (ICU), almost 3-fold more than among immunocompromised patients with GIT CMV [36]. The seriousness of the disease is highlighted by the possible need for surgery (in over 10%), long duration of hospital stay (median, 3 weeks), and potential mortality (over 20% in one month). Nevertheless, 25% had a milder disease course and recovered with supportive treatment alone. Spontaneous remission was reported in 24–54% (median, 32%) of 90 patients in three different series reported since 2005 [38]. One last intriguing and clinically important feature was the frequent absence of ‘typical’ CMV-suggestive features such as a shift to the right in the WBC, hepatitis, and even fever.

In addition to upper- or lower-GIT bleeding, which can be severe, a survey of abundant case reports published in recent years uncovers many potential complications of GIT CMV. They include surgical emergencies such as perforation (when ulcers are deep), obstruction (when inflammation causes stricture formation), or megacolon; and diagnostic challenges such as concurrent *clostridium difficile* pseudomembranous colitis, EBV reactivation, or associated inflammatory bowel disease (see below). Importantly, GIT CMV may mimic GIT malignancy, ischemic colitis, and other conditions, requiring a high index of suspicion for a timely and correct diagnosis. A particular form of CMV gastropathy or ileitis may present with more severe hypoalbuminemia and generalized edema due to protein-losing enteropathy (PLE) [39]. The mainstay of additional, often-needed treatment is intravenous ganciclovir or its prodrug—oral valgancicovir. Both have good bioavailability and a therapeutic efficacy of >80% has been achieved [40].

Selected illustrative case reports of CMV esophagitis, gastritis, ileitis, appendicitis, colitis, and proctitis [41,42,43,44,45,46] highlight several recurring themes. First, that any segment of the entire gastrointestinal tract can be affected by CMV. Second, that the involvement of more than one part is not uncommon. And third, that clinical disease can be severe and life-threatening.

Finally, a complex and incompletely understood relationship exists between CMV colitis and inflammatory bowel disease [47]. Often, IBD patients are especially susceptible to superimposed CMV infection of the colon, regardless of their inflammatory disease activity or immunosuppressive medications [48]. Moreover, refractory ulcerative colitis should be evaluated for CMV, since the appropriate antiviral treatment may obviate the need for colectomy [49]. However, both concurrent diagnoses of CMV colitis and IBD colitis have been reported; intriguingly, CMV colitis may later be followed by the development of distinct, proven IBD [50].

#### 3.2.2. Hepatic and Pancreatic

While acute hepatitis is almost the *sine qua non* of active CMV infection [25,27,30], it is benign and self-limited in the majority of cases, although overt jaundice (often mild) was reported in 3% of 115 immunocompetent hospitalized patients [27], and the associated inflammatory response may be severe enough to cause normocytic anemia (e.g., Hb 11 gr/dL), hypoalbuminemia (e.g., 2.9 g/L), and markedly increased ferritin levels (e.g., 2640 ng/mL) [51]. The serum levels of transaminases are typically in the hundreds and alanine aminotransferase is often elevated to a higher extent than aspartate aminotransferase (e.g., SGPT 495 vs. SGOT 233 U/L) [52]. Peak SGPT levels of ~2900 U/L have been described [52]. Cholestatic liver enzymes are often increased as well, but to a lesser extent. Nevertheless, normal liver enzymes cannot exclude acute CMV, especially when the presentation is predominantly in the GIT or neurologic [25,36]. The decision to start antiviral therapy [52] or withhold it [53] needs to be patient-tailored; there are neither trials nor guidelines in that regard. A concurrent involvement of an additional organ (e.g., the CNS) may greatly influence the decision to commence treatment. Notably, in extremely rare instances, fulminant hepatitis may develop, mandating emergency liver transplantation. In other isolated cases, a predominantly cholestatic variant or the development of ascites due to portal hypertension may occur.

The causes of acute pancreatitis are gallstones or alcohol in the majority of cases [54]. Strict diagnostic criteria have linked acute pancreatitis with well-documented viral infection, including CMV [55], but these cases are few and far between, even when immunocompromised patients are included: not more than 209 cases had been identified in a recent review of the literature and CMV was involved in just 25/209 (12%) [56]. Nevertheless, in the right clinical context, this is an important, though extremely rare diagnosis to consider, possibly related to gastrointestinal CMV affecting the duodenal papilla or the common bile duct (‘CMV cholangiopathy’) or perforating the posterior gastric wall [57,58,59].

A detailed summary of all reported gastrointestinal, hepatic, and pancreatic manifestations is presented in Table 1.

**Table 1:** The spectrum of gastrointestinal, liver, and pancreatic disease in CMV-infected immunocompetent patients.

**A. Gastrointestinal Involvement** * (Relatively Common)
Esophagitis
Gastritis
Duodenitis
Ileitis (and enterocolitis)
Appendicitis
Colitis (and proctocolitis) ^
Proctitis
Splanchnic vein thrombosis (splenic vein; portal vein; mesenteric vein; hepatic veins) (probably rare but should be considered)
**B. Liver and Pancreaticobiliary involvement** (liver involvement: common)
Hepatitis ± jaundice (common) (predominantly hepatocellular; rarely cholestatic)
Hepatomegaly (common, median, 9%)
Fulminant hepatitis * (rare)
Hepatitis presenting with ascites (high gradient) and portal hypertension (rare)
Acute pancreatitis (probably rare)
Cholangitis (rare)

* Associated mortality has been reported. ^ May be associated with inflammatory bowel disease (IBD)—preceding, concurrent, or complicating its course (see text).

### 3.3. Severe CMV-Associated Neurologic and Ocular Manifestations in Immunocompetent Hosts

#### 3.3.1. Neurologic

Severe cytomegalovirus infection in immunologically normal patients can take several forms and is important to recognize. In Eddleson’s original review of the worldwide literature comprising thirty-four patients [25], seven had neurological involvement together with other severe disease (e.g., liver, lungs) without including retinal involvement, and ten had isolated CNS disease (untreated in four); all survived. Rafailides, who retrieved 89 articles on 290 immunocompetent patients, found that central nervous system (CNS) disorders constituted the second most frequent manifestation of CMV [26], and Wreghitt notes confusion in 4/18 (22%) hospitalized patients [28]. What forms does severe CMV-associated neurological disease take?

*The first* possible neurological presentation of CMV infection in immunocompetent patients is that of acute viral meningitis, meningoencephalitis, or encephalitis. Polymerase chain reaction (PCR) analyses of the cerebrospinal fluid (CSF) have revolutionized the diagnosis of viral CNS infections, especially those caused by human herpesviruses [60]. Generally, CMV is the least encountered of all herpesviruses in cerebrospinal fluid (CSF) samples. In two large studies of CSF samples of patients suspected of CNS infection, 136 were positive for herpesviruses by PCR: 60 cases of herpes simplex virus (HSV), 26 of varicella zoster virus (VZV), 23 of Epstein–Barr virus (EBV), 10 of human herpes virus 6 (HHV6), and only 2 of CMV [61,62]. Among the CSF samples of 146 patients with acute herpesvirus meningitis or meningoencephalitis, 39 were found positive for human herpesvirus DNA: 25 were identified as VZV or HSV, and CMV was not found [63]. Nevertheless, rare as it may be, CMV is a confirmed cause of viral CNS infections in immunologically normal adults. A handful of reports have been identified and CMV should therefore be included in the differential diagnosis of patients presenting with encephalitis, meningitis, or meningoencephalitis [64,65,66]. The clinical presentation is that of an acute or sub-acute development of fever, headache, nausea, nuchal rigidity, drowsiness, lethargy, confusion, dysphasia, upper motor neuron or cranial nerve signs, or epileptic seizures, in any combination. No concurrent CMV-associated changes and neither pathology nor mortality have been reported [25]. The frequent absence of lymphocytosis or hepatitis should be noted [63,65].

*The second* important presentation of CMV-associated neurological presentation in normal hosts is that of demyelinating syndromes of the spinal cord, optic nerve, or both. A patient who presented with CMV-induced severe spastic paraparesis due to transverse myelitis (with normal blood counts and liver enzymes) was treated with ganciclovir but later developed symptoms and magnetic resonance imaging (MRI) detected white matter changes suggestive of disseminated demyelination (ADEM) [67]. We have found single case reports on 12 patients with substantiated CMV transverse myelitis. They presented with progressive lower limb weakness, numbness, and possibly sphincter dysfunction, and with or without fever, lymphocytosis and increased liver enzymes. Diagnosis was based on typical MRI findings, blood serology, and the detection of CMV DNA in CSF. Early initiation of high-dose glucocorticoids was deemed important, and prognosis was good [68,69]. The only fatality reported was due to an associated CMV pneumonitis. Another, distinct type of CMV-induced neurological manifestations are neuromyelitis optica (NMO) spectrum disorder demyelinating syndromes, which present as painless visual loss due to optic neuropathy with or without extensive longitudinal transverse myelitis [70,71]. An association with another herpesvirus, i.e., preceding VZV infection, has been reported [72]. The pathogenetic role of specific (CMV-associated?) NMO-IgG or aquaporin-4 autoantibody (AQP4-IgG), directed against the principal CNS water channel aquaporin-4, remains to be elucidated.

*Finally*, primary CMV infection may be followed by a peripheral nervous system demyelination: Guillain–Barre syndrome (GBS) [73]. In a prospective study of 506 GBS patients, 12.4% were associated with active CMV infection by IgM detection, IgG avidity, and frequent (62%) CMV DNA in plasma at hospital admission [74]. A 154-patient case–control study yielded similar results [75]. Most patients were previously healthy and young (median age 32 years), and the pathogenesis involved an aberrant immunological post-infectious response to CMV (or other agents, in particular *Campylobacter jejuni*) and not a direct infection [76]. Nevertheless, concurrent ‘silent’ hepatitis, when present, may support antecedent CMV infection [77].

#### 3.3.2. Ocular

Unlike immunocompromised patients, in whom necrotizing retinitis is the most common ocular manifestation of CMV infection, immunocompetent individuals predominantly develop anterior uveitis [78]. This entity has a spectrum of clinical manifestations including endotheliitis, iris atrophy, and anterior uveitis, which may present in an acute form as Posner–Schlossman syndrome (PSS) or, more chronically, as Fuchs uveitis syndrome (FUS) [79]. Rothova el al. undertook a retrospective cohort study of 1075 immunocompetent patients with uveitis. Only 125/1075 had infectious causes (11.6%), about half of them viral, but CMV was identified in 3 patients only [80]. Patients usually present with eye pain, conjunctival injection, and blurring of vision, sometimes associated with halos and ipsilateral headache [79]. Diagnosis is usually made by obtaining aqueous fluid through anterior chamber paracentesis. Antibody analysis and, in particular, PCR are essential for detecting CMV DNA and for accurate diagnosis. Currently, CMV anterior uveitis is increasingly recognized and treated worldwide [81]. CMV anterior uveitis is mostly unilateral, hypertensive (associated with secondarily increased intraocular pressure), and may be recurrent, but offers a fairly good long-term visual prognosis [81]. CMV retinitis or retinal necrosis have also been reported in immunocompetent cases, but these patients also had anterior chamber inflammation [78].

A detailed summary of all reported neurologic and ocular manifestations is presented in Table 2.

**Table 2:** The spectrum of neurologic and ocular disease in CMV-infected immunocompetent patients.

**Neurologic Involvement**
Viral encephalitis ^
Viral meningitis ^ (mononuclear pleocytosis)
Meningoencephalitis ^
Optic neuritis (bilateral papillitis) (rare)
Neuromyelitis optica (rare) (optic neuritis and longitudinal extensive myelitis)
Longitudinal extensive myelitis (rare)
Transverse myelitis (rare)
Myeloradiculitis (rare)
Guillain–Barre syndrome (relatively common)
Cerebral sinus vein thrombosis (see Hematology) (rare)
Horner syndrome, unilateral (rare)
Myotonia, ‘essential’ (rare)
**Ocular involvement**
Anterior uveitis (mostly unilateral, with increased intraocular pressure)
Intermediate uveitis
Glaucoma ^^, uncontrolled
Chorioretinitis ^^
Ac. retinal necrosis ^^
Papillitis (see neurology)
Horner syndrome, unilateral (rare)

^ Prevalence not reported but may not be rare and the diagnosis should be considered. ^^ Associated with anterior chamber and vitreous inflammation. Other than uveitis, other ocular complications are rare (and secondary).

### 3.4. Severe CMV-Associated Hematologic Manifestations in Immunocompetent Hosts

Although the list of different hematological syndromes reported in severely CMV-infected immunocompetent patients is an extensive one, a closer inspection reveals just two major phenotypes: cytopenias and venous thrombosis. Their presentation, however, may be highly varied.

#### 3.4.1. Cytopenias

Before looking at cytopenias in severe CMV infections, the characteristic blood count of ‘CMV mononucleosis’ in immunocompetent patients should be reviewed. The predominant feature is lymphocytosis, with prominent atypical lymphocytes seen in 78–98% of cases and accompanied by normal or slightly elevated white blood cell (WBC) count [28,82,83]. Anemia is common (up to 67%) [84], but is usually mild, normocytic, and due to the inflammation (anemia of inflammatory disease) [51]. Finally, thrombocytopenia is common in viral infections due to multiple inflammation-associated factors [85], and CMV is no exception. Thrombocytopenia is usually non-severe and asymptomatic; it was observed in 2–3% of 150 patients from France [82,83] and in 12.5% of 54 American patients [32,84]. All these changes are transient and self-limited, although they may take weeks to normalize. Splenomegaly is detected in up to a third of the patients (vs. hepatomegaly in ~10%), and ~20% have lymphadenopathy. Together with the common prolonged fever (95%), night sweats (20%), and weight loss, a lymphoproliferative disorder with ‘B symptoms’ may erroneously be suspected. This is especially true since herpesviruses may present clinically with Pel–Ebstein type of fever [86,87].

Symptomatic cytopenias are considerably more unusual. A recent report identified five new cases of symptomatic CMV-associated thrombocytopenia and collected twenty-five more patients from the literature [88]. The mechanism is likely to be immune-mediated (i.e., immune thrombocytopenic purpura, ITP), and in some cases, platelet-associated immunoglobulins were demonstrated with normal bone marrow megakaryocytes. Other postulated mechanisms include a direct effect of CMV on bone marrow cells and the downregulation of hematopoietic growth factors. Additional organ involvement by CMV, in particular hepatitis, is commonly associated. While the response rate to first-line glucocorticoids is partial (1:3), 75–82% respond to antiviral treatment or thrombopoietin receptor agonists. Other notable (but rare) CMV-induced cytopenias include thrombotic thrombocytopenic purpura, hemophagocytic syndrome, disseminated intravascular coagulation, and hemolytic anemia. In addition, polyclonal hypergammaglobulinemia and diverse induced autoantibodies and proteins, including antinuclear antibodies (ANAs), rheumatoid factors (RFs), anti-red cell antibodies (Coombs), antiphospholipid antibodies (APLAs), paraproteins, monoclonal gammopathy of undetermined significance (MGUS), cryoglobulins (in one case report, associated with decreased complement levels and renal disease), and cold agglutinins, may be detected, but they rarely cause clinical disease before disappearing (see Table 3).

#### 3.4.2. Vascular Thrombosis

Regarding venous thrombosis complicating acute CMV infection in immunocompetent hosts, approximately 125 cases were found in a literature review and systematic review [89], and the association has been well studied. In two retrospective studies of immunocompetent patients hospitalized with venous thromboembolism, 10/1007 (1%) or 5/262 (1.9%) had acute CMV infection [90,91]. Importantly, 9:10 patients (or 66% in another study) had an underlying thrombophilic factor other than CMV infection, usually contraceptive pill use or hereditary factor V Leiden mutation [89]. Transient, CMV-induced antiphospholipid antibodies (APLAs) may be an additional risk factor [88]. When unprovoked venous thromboembolism (VTE) patients were compared to controls, the adjusted OR for active, IgM-positive CMV was 5.26 (95% CI 1.35–20.8, *p* = 0.017) [92]. Conversely, active CMV was independently associated with VTE development (OR 2.49, 95% CI 1.53–4.06, *p* < 0.0001) in a large, prospective community study [93]. Any venous vascular bed can be involved, and though deep vein thrombosis and pulmonary embolism (DVT/PTE) are the most frequent VTEs, splanchnic vein thrombosis (splenic vein thrombosis; portal vein thrombosis; mesenteric vein thrombosis; hepatic vein thrombosis) [94], infarction of the spleen [95], small-bowel infarction, portal hypertension, and even cerebral sinus vein thrombosis have been reported. An association of CMV with *arterial* thromboembolisms has been postulated but remains controversial [93]. A detailed summary of all reported hematologic manifestations is presented in Table 3.

**Table 3:** The spectrum of hematological involvement in CMV-infected immunocompetent patients.

Splenomegaly (common, median, 23%)
Lymphadenopathy (common, median, 19%)
Splenic infarction * (rare)
Spontaneous splenic rupture * (rare)
Anemia of inflammatory (‘chronic’) disease (common)
Thrombocytopenia (common) (may be severe and refractory, rare)
Thrombotic thrombocytopenic purpura (TTP)—severe thrombocytopenia and microangiopathic hemolytic anemia (MAHA) (rare)
Multiple microthrombi (with reduced ADAMTS13, increased VWF) (rare)
Hemophagocytic syndrome (HPS) (rare)
Hemolytic anemia (may be severe, Coombs-negative) (rare)
Cold agglutinins (prevalence not reported, probably rare)
Disseminated intravascular coagulation (DIC) (rare)
Leukopenia and thrombocytopenia, severe symptomatic * (rare)
Pancytopenia, severe symptomatic * (rare)
Bone marrow changes (myeloid dysplasia, multiple lymphoid nodules, infrequent hematopoietic precursors) (rare)
Deep vein thrombosis (DVT)/pulmonary embolism (PTE) * #
Splanchnic vein thrombosis (splenic; portal; mesenteric; hepatic veins) * #
Cerebral sinus vein thrombosis # (rare)
Hypergammaglobulinemia, polyclonal (common)
Ferritin > 1000 ng/mL (prevalence not reported)
Cryoglobulinemia (hypocomplementemia; ac. kidney injury) (rare)
Paraproteinemia (rare)
Monoclonal gammopathy of uncertain significance (MGUS), transient
Autoantibodies (antinuclear antibodies, rheumatoid factor, Coombs), transient (may be common, but not associated with clinical disease)
Antiphospholipid antibodies (APLAs), transient ± infarction (e.g., of spleen) (rare)
Cutaneous (leukocytoclastic) vasculitis (rare)
Systemic vasculitis (rare) (Wegener’s granulomatosis, polyarteritis nodosa)
EBV immunoreactivation (probably unusual)

* Associated mortality has been reported at 5–22% in CMV-associated thrombosis. # An additional hypercoagulability factor is commonly present. Prevalence has not been reported but may not be rare and CMV should be considered in this setting.

### 3.5. Severe CMV-Associated Pulmonary and Cardiac Manifestations in Immunocompetent Hosts

#### 3.5.1. Pulmonary

The two prime types of pulmonary involvement in CMV are pulmonary thromboembolism (discussed under Hematology) and acute CMV pneumonitis. They remain a rare manifestation, described in isolated case reports, although there are insufficient data to determine their exact frequency. In Bonnett’s et al. retrospective study of 115 hospitalized CMV patients, just 2 had pneumonitis (1.7%) [27]. A 2012 review of the literature found 13 cases (including 9/34 collected by Eddleston) of patients who had an additional organ involvement in all but 1 case [25,96]. Another <10 case reports have been published since. The clinical presentation is indistinguishable from other viral pneumonias. Other than the frequent (but not universal) co-occurrence of hepatitis, lymphocytosis, and possibly an additional organ’s involvement due to CMV, the physical examination and even the chest radiograph, WBC, and CRP may be normal, even though patients present with fever, cough, and dyspnea with associated hypoxemia [97]. Chest CT better defines diffuse interstitial infiltrates, found predominantly in the lower lobes. CMV diagnosis is as described above, although additional viral and other causes (e.g., SLE) have to be excluded [98]. Close monitoring and a light finger on the trigger of initiating specific treatment (e.g., oral valganciclovir) is needed [97,99]. The course may either be self-limited without treatment or deteriorate to an acute respiratory failure, SIADH, or diffuse alveolar hemorrhage [100].

#### 3.5.2. Cardiac

Acute CMV myocarditis or perimyocarditis is the best documented cardiac complication and can appear either as isolated (with a normal blood count and normal liver enzymes) [101] or, more commonly, as associated with hepatitis, encephalitis, or other organ involvement [25,102]. Fever is almost always present. Its severity is obvious when looking at the frequent development of overt, sometimes severe, heart failure [103] and the molecular studies conducted on the hearts of 17 patients who died of myocarditis: cytomegalovirus nucleic acids were found in 15 of them [104]. Only one of these patients was immunocompromised. Despite the danger of mortality, most of the patients reported fared well, some of them even without specific treatment of CMV [102], and their frequently compromised left ventricular ejection fraction returned to normal. It is likely that subclinical myocardial involvement occurs in more patients but remains unrecognized. Electrocardiography, echocardiography, and cardiac MRI provide the best diagnostic information, as do cardiac muscle enzymes (troponin), which are frequently increased [105]. Myocarditis is an infective and inflammatory process that may involve not only the myocytes, interstitium, and vascular elements of the heart muscle [105], but also the pericardium [106]. In these cases, pleuritic chest pain is usual, and pericardial effusion may occur. In fact, in a study of 53 consecutive patients admitted with large pericardial effusion, viral infection was diagnosed in 8 (14%); the second most common etiology after malignancy and CMV was the most common microorganism, identified in 3 patients [107]. When pericardial fluid is available for analysis, molecular techniques (PCR) significantly increase the diagnostic yield [108], though CMV was not identified in 57 samples. To complete the discussion, the potential long-term effects of myocardial CMV infection must be considered. CMV genome persistence in the myocardium of patients with dilated cardiomyopathy is well-established, albeit rare [109]. In endomyocardial biopsies, the viral genome was frequently detected by PCR and histo- and immunohistochemistry (32/42 patients, 76%) and CMV was the most frequently identified virus found (followed by Coxsackie B virus), suggesting a causative link. These cases of ‘idiopathic’ dilated cardiomyopathy likely represent the final outcome of subclinical and undiagnosed CMV myocarditis suffered years prior.

A detailed summary of all reported pulmonary and cardiac manifestations is presented in Table 4.

**Table 4:** The spectrum of pulmonary and cardiac involvement in CMV-infected immunocompetent adult patients.

Pulmonary
Pneumonitis * (prevalence not reported, probably rare; chest X-ray may be unremarkable)—may progress to acute respiratory distress syndrome (ARDS) and acute respiratory failure (rare)
Hyponatremia secondary to syndrome of inappropriate antidiuretic hormone (SIADH) (rare)
Pulmonary embolism * (PTE) (see Hematology)
Pulmonary veno-occlusive disease? (rare)
May rarely complicate interstitial lung disease (ILD) *
**Cardiac ^**
Myocarditis * (may lead to heart failure) (prevalence not reported)
Pericarditis, pericardial effusion (prevalence not reported, probably rare)
Perimyocarditis *
Dilated cardiomyopathy *
Incident myocardial infarction* (OR 2.1) or congestive heart failure * (OR 3.8) within <5 years of active CMV according to 1 case–control study from Korea

* Associated mortality has been reported. ^ Prevalence is unknown but CMV should be considered in this setting.

### 3.6. Severe CMV-Associated Cutaneous Manifestations in Immunocompetent Hosts

The more severe cutaneous manifestations, such as thrombocytopenic petechiae or purpura, are quite rare (see under Hematology), whereas cutaneous vasculitis is more encountered and is even mentioned in a few series [27,29,109]. However, rashes (usually maculopapular, possibly following empiric ampicillin treatment) in patients with severe CMV of other organs are more common, and despite their nonspecific nature, may help direct attention to the possibility of CMV infection in immunocompetent hosts. Prevalence varied from 6/82 patients (7.3%) in a mixed group of patients, of whom 40% had been hospitalized [84], to 4/38 (10.5%) severely ill CMV-infected patients [30] and 40/199 (20%) in three studies from France [82,83], and up to 19% in a smaller US series [32]. Other, more specific cutaneous manifestations are listed in Table 5.

These and additional varied manifestations are summarized in Table 5.

**Table 5:** The spectrum of severe systemic and mucocutaneous involvement and the involvement of other organs in CMV-infected immunocompetent adult patients.

Severe Systemic Symptoms
Severe symptomatic illness with any combination of protracted fever (low-grade or high; possibly cyclic); sweats (especially night sweats); anorexia; prominent weight loss; fatigue; lassitude; and hepatitis (possibly with jaundice). May be severe enough or of uncertain cause to warrant hospitalization (common). May be protracted and last ≥8 weeks, with fatigue/lassitude persisting long after normalization of blood tests.
**Mucocutaneous**
[Jaundice, in 3–22%] (see: Liver) (infrequent but not unusual)
Rash secondary to ampicillin (similar to EBV mononucleosis) (common)
Rash, nonspecific (in 19%, median) (common)
Thrombocytopenic purpura (rare) (see: Hematology)
Cutaneous vasculitis (rare)
Skin ulcers (rarely complicate drug hypersensitivity syndromes)
Erythema multiforme (rare)
Vesicular and pustular eruption (rare)
Gianotti–Crosti (cutaneous eruption) syndrome (rare)
**Other**
Polymyalgia rheumatica-like syndrome (rare)
Rhabdomyolysis, severe (may affect respiratory muscles) (rare)
Ac. cervicitis and vulvovaginitis (rare)
Hematuria due to ureteritis (rare)
Ac. bacterial sinusitis; CMV may be precipitating factor (rare)
Incident type 2 diabetes within <5 years of active CMV according to 1 case–control study from Korea (5.6% vs. 2.2%) (requires confirmation)

### 3.7. CMV Infections in Special Populations

#### 3.7.1. Critically Ill Patients

Extensive research has been devoted to CMV infection (i.e., primarily reactivation) in the intensive care unit (ICU), including to its prevalence, impact, and treatment. CMV has been increasingly recognized in critically ill immunocompetent patients. Many were previously infected (seropositivity rate of 71%) and active CMV infection was common: in a systematic review and meta-analysis of 18 studies and 2398 patients, 27% (95% CI 22–34%) of immunocompetent patients were found to be infected [110]. Sepsis and mechanical ventilation were the strongest risk factors identified [111]. Compared with patients without CMV infection, the clinical course of patients who developed CMV infection in the ICU was more severe (longer mechanical ventilation, longer ICU stay) and their mortality was doubled (OR 2.16, 95% CI 1.70–2.74) [110]. CMV infection occurs mostly between day 4 and day 12 of ICU admission, and reactivation with viremia was commonly observed when looked for [112]. These observations were confirmed across myriad conditions such as sepsis (highest incidence of CMV infection), critical heart surgery patients, after major surgery, in acute respiratory distress syndrome, and severe burns [110,113,114,115], and it can develop in seronegative patients as well, representing a primary infection. Clinical manifestations can be subtle and difficult to discern against the background of these patients’ severe multi-organ disease. For example, lower gastrointestinal bleeding could be due to CMV colitis [116]. However, despite its prevalence and impact, there is no solid evidence yet on the cost-effectiveness of screening for CMV infection in the ICU or on the initiation of antiviral treatment unless specific CMV-induced organ involvement is documented.

CMV reactivation should also be considered in acutely ill immunocompetent inpatients with serious illness in the department of medicine. A pro-inflammatory state has been postulated as a key factor in triggering CMV reactivation. Thus, CMV DNA was detected in the blood of 6/80 (7.5%) patients with community-acquired pneumonia (CAP), which is a notable finding, although these patients’ CMV was not treated, and their clinical course was similar to patients in whom CMV DNAemia was not detected [117]. Of special interest is the well-documented evidence of CMV reactivation in patients hospitalized with COVID-19. In one study, CMV DNA was discovered in 88/431 (20.4%) ICU patients critically ill with COVID-19 [118]. No effect of CMV reactivation on the clinical course was established. Among less severely ill COVID-19-infected patients, 16/140 (11.4%) had CMV reactivation, clinically unsuspected by their physicians [119]. Nevertheless, a negative impact of CMV reactivation in immunocompetent patients under similar circumstances cannot be ruled out (i.e., 2.5-fold increase in ICU mortality, prolonged duration of mechanical ventilation, and increased length of ICU stay), as reported in a meta-analysis of 22 studies published in 2017 [120]. In another study, plasma CMV was assessed by PCR among 132 consecutive patients admitted for acute heart failure, another condition where inflammatory mediators such as cytokines have an established role. CMV DNAemia was detected in 11/132 (8.3%) of patients, albeit at a low level of <100 copies/mL [121]. When evaluated by clinicians blinded to CMV results, deaths and readmissions were significantly increased in the CMV-positive group (HR 4.39 95% CI 2.02–9.52).

#### 3.7.2. Pregnancy

CMV infection in pregnancy is usually asymptomatic, as in the general population, and is diagnosed either by seroconversion, IgM positivity with low IgG avidity, or a 4-fold increase in IgG antibody titer. It has to be routinely monitored due to the potentially devastating effects of congenital CMV on the newborn [122]. The focus on neonatal outcomes notwithstanding, pregnant women can acquire severe CMV infection. Indeed, pregnant women are represented in the series of patients with severe CMV [25,28] and in case reports, but their course is generally favorable and the complexity of the immune mother–fetus interaction does not necessarily qualify pregnancy as a state of immunological weakness or suppression [123].

#### 3.7.3. Elderly Patients

Latent CMV infection is material in the development of immunosenescence, and immunosenescence is important in age-related CMV reactivation, exhibiting a significant bidirectional relationship [9,10]. Reactivation according to IgM seropositivity varies from 15% in young persons to 63% in those over 60 years [124,125]. These percentages vary greatly in different populations. However, reported patients with severe CMV infection are often not elderly. Among Eddleson’s series of 34 patients, only 2 were over 65 [25] and only 2 of 18 hospitalized patients were over 60 [28]. Likewise, none of Nolan’s American patients were over 65 [32] and the mean age in a French series was 39 years [81]. A notable exception is the important group of patients with GIT involvement, who were often in their 60s or even 70s [35,36,37,38]. Nevertheless, clinically important CMV reactivation and primary infection do occur in older adults and may be underrepresented in the literature owing to selection bias, a well-known concern in research on older adults [126]. CMV in older adults appears to cause a more severe mononucleosis-like syndrome with multi-organ involvement requiring antiviral treatment [31]. Increased mortality has not been reported.

#### 3.7.4. Inflammatory Bowel Disease (IBD) Patients—See under Gastroenterology [47,48,49,50]

### 3.8. Long-Term Impactful Consequences of Latent CMV Infection

Although not really within the scope of this chapter, we will briefly discuss the possible long-term effects that have been associated with past CMV infection (i.e., CMV latency) in view of the astounding worldwide seroprevalence [127] and potential implications of the lifelong infection maintained by CMV in immunocompetent hosts (Table 6). These postulated consequences, many years after an infection contracted early in life, are especially intriguing.

A study from Johns Hopkins prospectively examined a cohort of 635 community-living women in their eighth decade of life, finding that 85% had past CMV infection, and categorizing them into four groups according to quartiles of the titer of CMV antibody concentration [128]. They found that women in the highest quartile had a significantly greater incidence of frailty (HR 3.46, 95% CI 1.45–8.27) and mortality (HR 3.81, 95% CI 1.64–8.83). The significant risk of mortality persisted after adjustment for potential confounders (HR 2.79 for the highest quartile). This is supported by a later prospective study of a European cohort of 13,090 participants, aged 40–79 at recruitment [129]. A total of 59% had CMV IgG antibodies and their age- and sex-adjusted mortality rates were significantly higher compared to seronegative participants. Although the difference was not striking (14.2 vs. 12.4 per 1000 person years), they increased across thirds of IgG antibody titers.

These data, interesting as they may be, need to be interpreted with caution. In some studies, CMV had a *positive* contribution to the immune response: for example, an enhanced antibody response to influenza vaccines was identified in seropositive young adults and was not seen with EBV [130]. Aging had a predominantly negative effect on most immune parameters [131], and alternatively, frailty could be the prime event driving CMV reactivation and, thus, the observed increase in CMV antibodies. Therefore, the hypothesis that CMV may drive immunosenescence and affect immune reactivity may be questioned by recent evidence [131].

Infectious agents, mostly viruses (including CMV), have been investigated in the context of Alzheimer’s disease since a key role of neuroinflammation in neurodegenerative diseases is increasingly recognized [132]. In this context, both CMV seropositivity and a higher IgG antibody response were associated with lower cognitive function among 5617 adults aged 65 years or more included in the US-based, nationally representative Health Retirement Study (HRS) [133].

Many studies have been devoted to accumulating data supporting the possible role of CMV in the development and expression of atherosclerosis and atherosclerotic plaques [134,135]. Data were derived from laboratory research, animal models, epidemiological studies, and the detection of CMV DNA in plaques. Multiple underlying mechanisms are involved and remain unclear; however, CMV interaction with vascular endothelial cells and smooth-muscle cells, as well as monocytes, macrophages, and T-cells, is being studied and information is accumulating, including the elucidation of its potential clinical impact. For example, after a mean follow-up of 12 years of 12,574 participants without ischemic heart disease (58% seropositive for CMV), significantly more seropositive patients in the highest-antibody group had incident ischemic heart disease (IHD) after adjustment for classical risk factors and other confounders (HR 1.21) [136]. A similar study from Oxford however, found no such association [137], and the issue remains controversial. Nevertheless, an elegant meta-analysis of studies up to 2016 that included 10 articles and 34,564 patients supports antecedent CMV infection as a significant risk factor in future cardiovascular events (cardiac and CNS) and death (RR 1.22, 95% CI 1.07–1.38, *p* = 0.002) [138]. In this context, a relatively short-term effect of CMV infection was reported in a single case–control study from Korea. When 667 CMV patients (with active disease and not detected via serology) were compared to matched controls in a 1:10 ratio, they had significantly more incident myocardial infarctions (OR2.1, 95% CI 1.0–4.5) and congestive heart failure (OR 3.8, 95% CI 2.1–6.8) when followed between 2010 and 2015 [139].

Lastly, CMV seropositivity was identified as a significant risk factor for contracting a reactivation of a latent infection of another herpesvirus, herpes zoster (OR 3.06, 95% CI 1.32–7.04), which is an intriguing finding [140].

Though more associations are reported in Table 6, a solid database supports the role of CMV in fueling a pro-inflammatory state and the progression of immunosenescence [10,118], while the evidence for CMV as one of the environmental factors triggering autoimmune diseases is much less convincing [141].

These and other associations are summarized in Table 6.

**Table 6:** The spectrum of postulated long-term adverse consequences associated with past CMV infection in immunocompetent adult patients.

In a population-based study, seropositivity was associated with all-cause mortality.
In older women, seropositivity was associated with an increased risk of mortality and frailty.
In the large, nationally representative Health and Retirement Study (HRS) in the US, seropositivity (and a higher IgG antibody response) was associated with lower cognitive function.
In a population-based study, seropositivity was associated with incident ischemic heart disease.
In a UK population study, seropositivity was associated with an increased risk of herpes zoster (OR 3.06, 95% CI 1.32–7.04).
In women recently diagnosed with breast cancer, seropositivity was associated with increased fatigue.
In schizophrenia spectrum disorders, seropositivity was associated with a smaller cortical surface and (in another study) a lower intelligence quotient (IQ).
Immunosenescence (bidirectional relationship, see text).
Autoimmune diseases? (systemic lupus erythematosus, Sjogren’s syndrome)
Atherosclerosis and plaque progression?

## 4. Conclusions

In conclusion, CMV is a ubiquitous DNA virus that can cause disease, mostly by primary infection or the reactivation of latent infection in up to 100% seropositive individuals. In immunocompetent patients, CMV infection is asymptomatic or mild and self-limited in the majority of cases, as opposed to immunocompromised hosts, in whom invasive CMV infections are quite often encountered. However, we have shown that despite immunocompetence, CMV can cause a very large variety of clinical syndromes in any part of the gastrointestinal tract (the most common pattern), the central (or peripheral) nervous system, and the eyes, as well as causing hematological, pulmonary, cardiac, and cutaneous disease. Not uncommonly, more than one system is involved, and though the disease is often self-limited, treatment with intravenous ganciclovir or oral valganciclovir may be required. In isolated cases, fatalities may occur, and long-term complications have been postulated. Thus, CMV infection should be considered in the differential of myriad syndromes in non-immunocompromised patients. Some populations, such as critically ill patients in intensive care units, pregnant women, elderly patients, and those with inflammatory bowel disease, may be more susceptible. Moreover, the potential of past, latent CMV infection to be associated with significant morbidity and all-cause mortality years later without reactivation is intriguing and requires further study. All these data indicate the great importance of developing a vaccine against CMV, which will hopefully become available in the foreseeable future. Currently, using messenger RNA vaccine technology and the immunogenic pentamer complex of five surface proteins rather than glycoprotein B may overcome years of frustrating efforts [142,143]. Meanwhile, a solid diagnosis of active CMV infection can be quickly established (or ruled out) by widely available serology tests and PCR amplification, and clinicians in all disciplines need to be more aware of the diverse guises of CMV infection and remember to consider it in any host, including an immunocompetent one.

## Data Availability

The original contributions presented in the study are included in the article, further inquiries can be directed to the corresponding author.
